# Knowledge, attitudes, and practices related to adult vaccination among adults and healthcare professionals across Mexico

**DOI:** 10.1371/journal.pone.0348625

**Published:** 2026-05-21

**Authors:** Erika Hurtado-Salgado, Eduardo Lazcano-Ponce, Martín Romero-Martínez, Efrain Alonso Terrazas-Medina, Salma Pacheco, Adriana Guzman-Holst

**Affiliations:** 1 Instituto Nacional de Salud Pública, Cuernavaca, Mexico; 2 GSK, Mexico City, Mexico; 3 GSK, Wavre, Belgium; Centers for Disease Control and Prevention, UNITED STATES OF AMERICA

## Abstract

**Background:**

Despite substantial evidence supporting the benefits of vaccination across all age groups, achieving high vaccine coverage in adults remains a global challenge. Limited disease knowledge and vaccine hesitancy contribute to this issue. To increase vaccination rates, we need to understand the gaps in knowledge, attitudes, and practices (KAP) among adults and healthcare professionals (HCPs). Our primary objectives were to describe the KAP regarding vaccine-preventable diseases and vaccination among adults and HCPs in Mexico, and to identify differences by sociodemographic factors.

**Methods:**

We conducted an observational cross-sectional survey study in three states in Mexico from July to October 2024. Two surveys were administered to adults aged ≥20 years and HCPs to capture outcomes of general vaccine and disease KAP, particularly for vaccine-preventable diseases such as herpes zoster (HZ), influenza, whooping cough, and respiratory syncytial virus (RSV).

**Results:**

A total of 1,169 adults from the general population and 228 HCPs were included. Among the population group, 95.6% acknowledged the importance of vaccines, and 87.6% knew adults could receive routine vaccines. Knowledge of vaccine-preventable diseases ranged from high for influenza (97.3%) to limited for RSV (16.1%) and HZ (39.4%). Fewer knew about RSV (10.1%) and HZ (18.6%) vaccines.

Only 23.5% of HCPs had recommended the HZ vaccine to older adults, while more did so for whooping cough (52.2%), influenza (96%). The main factors for HCPs to accept and recommend vaccinations were knowledge of the disease, complications, and vaccine efficacy and effectiveness. Also, 69.8% indicated that information about the disease was the most critical factor in vaccination recommendations.

**Conclusion:**

The findings underscore the need to strengthen health communication and education, and address barriers that impede access to vaccination, particularly for less well-known vaccines. Bridging the gaps between KAP is essential to improving adult vaccination coverage, thereby reducing vaccine-preventable diseases and enhancing public health.

## Introduction

Vaccination stands as one of the most effective public health achievements due to its ability to prevent a large number of diseases and deaths [1]. According to the World Health Organization (WHO), vaccines prevent between 2 and 3 million deaths each year. An additional 1.5 million deaths could be avoided with improved vaccination coverage worldwide [2]. Vaccination is important throughout the life-course, starting at infancy when life-saving vaccines may be given from birth, with some vaccines requiring boosters to ensure long-term or even lifelong protection, and continuing into adulthood, especially for people with comorbidities or at older ages when immunosenescence diminishes the immune response [3]. Vaccination programs have been especially successful in infants and children, while, vaccination coverage rates among adults are low, and increasing these rates remains a significant challenge [4,5]. Therefore, adult vaccination is an imperative of the Immunization Agenda 2030, a global initiative to reduce mortality and morbidity from vaccine-preventable diseases for everyone, everywhere [1,6].

In Mexico, the national immunization program (NIP) recommends and implements vaccines based on age groups and at-risk populations. The NIP includes two separate vaccination schemes for adults, one for 20- to 59-year-olds and another for ≥60-year-olds. Both schemes cover tetanus and diphtheria, seasonal influenza, and coronavirus disease 2019 (COVID-19) vaccinations. In addition, for individuals aged 20–59 years, a measles and rubella booster is included, and for individuals aged ≥60 years, pneumococcal vaccination is included [7]. The vaccines included in the NIP are provided free of charge. However, a 2022 national health and nutrition survey reported low vaccination coverage in adults. Among adults aged ≥60 years, 49.1% had received the seasonal influenza vaccine before the last winter season and 24.4% the pneumococcal vaccine in the last year. Meanwhile, 57.3% of adults aged 20–59 years had received a tetanus toxoid-containing vaccine in the previous ten years. These data indicate that a large proportion of adults are not protected against vaccine-preventable diseases [8]. While vaccination hesitancy is an important factor explaining low coverage rates in adults in the United States and Europe [9,10], it is not a major factor in Mexico. A systematic literature review of vaccination barriers in Latin America, including studies from Mexico, identified lack of awareness of diseases and their vaccines, unfavorable socioeconomic factors, and low education levels as common obstacles affecting vaccination coverage [11]. Targeted interventions to improve awareness of vaccine-preventable diseases and vaccinations among adults may increase vaccination coverage. To develop these interventions, we first need to understand the gaps in knowledge, attitudes, and practices (KAP) and the perceptions of adult vaccination among adults vulnerable to specific preventable diseases, as well as among healthcare professionals (HCPs) managing such diseases.

We conducted an observational cross-sectional study to describe the KAP around adult vaccination in adults and HCPs in Mexico. The primary objectives were to describe the knowledge, attitudes, perceptions, and practices of preventable diseases and vaccination among (1) adults aged ≥20 years; and (2) among HCPs. The secondary objective was to describe and identify differences in vaccination KAP among adults and HCPs by sociodemographic variables.

## Methods

### Study design

This observational cross-sectional study was conducted from 1^st^ July to 21^st^ October 2024. The target population consisted of two groups. For the first group, we aimed to recruit adults aged ≥20 years from the general population who attended primary healthcare units affiliated with one of the social security institutions (Instituto Mexicano de Seguro Social) in municipalities in Mexico State, Morelos, and Mexico City. For the second group, we aimed to recruit HCPs, such as general practitioners, primary care practitioners, specialists, and nurses, from the same geographic states and municipalities. Approval to conduct the study was obtained from the Institutional Review Board (Comité de Ética en Investigación, approval number 1916) at the Instituto Nacional de Salud Pública, Mexico. This study complied with all applicable participant privacy laws, and participants provided verbal informed consent. The consent was read and witnessed by the trained interviewer in the electronic survey tool, and a copy of the consent details given to each participant.

### Study population and inclusion and exclusion criteria

The sample sizes for the groups were estimated at approximately 1,200 adults aged ≥20 years of either sex, and 200 HCPs. The study employed cluster sampling for the adult population and convenience sampling for the HCP population. Details of the target population, sample calculation, and sampling procedure are described in the Supplemental Materials S1 Appendix. The inclusion criteria for the adult group were individuals aged ≥20 years who (1) accepted the invitation to participate in the survey; (2) provided a signed and dated informed consent form; and (3) were willing to comply with all study procedures and remain available for the duration of the study. Exclusion criteria were (1) an inability to read and write; (2) living in collective housing; and (3) not understanding Spanish. The inclusion criteria for the HCP group were (1) first contact physicians, nurses, and specialist physicians (2) who agreed to answer the survey.

### Survey data collection

Two surveys were carried out, one among adults (Survey A) and one among HCPs (Survey B). Both were approximately 30-minute quantitative surveys and consisted of four sections. They incorporated components of the Health Belief Model (HBM) for predicting health behavior and the Social Norms Model (SNM) [12–15], similar to our previous application and validation of the HBM and SNM in a maternal population [16]. Section 1 queried enrollment and demographic data. Section 2 queried general vaccine and disease knowledge, as well as knowledge of specific preventable diseases (herpes zoster [HZ], influenza, whooping cough, and respiratory syncytial virus [RSV]) and their corresponding vaccines in adults. Section 3 queried attitudes, beliefs, and perceptions of disease and vaccination regarding HZ, influenza, and RSV. Section 4 queried practice and utilization of vaccines against influenza and pneumococcal disease and evaluated motivation and behavior around vaccines not currently in the NIP (i.e., HZ, RSV). The HBM informed the main questionnaire, yet the constructs were further adjusted to the social and demographic context of the Mexican population. We divided the survey into sections based on the model constructs, yet we formulated the questions to be easier to understand for the general population, given low educational levels. Questions were also separated into two parts: first, knowledge of the disease, and then knowledge about the vaccine. The responses were operationalized and analyzed in the four main sections. Specifically, Section 3 queried attitudes, beliefs, and perceptions of disease and vaccination, with subcategories aligned with HBM concepts of susceptibility, severity, benefits, and barriers. The surveys were conducted in Spanish and consisted mostly of multiple-choice questions, with a few open-ended questions. They were administered by experienced, trained interviewers using electronic devices. The Research Electronic Data Capture platform monitored the number of surveys conducted daily.

### Outcome variables

The outcome variables included knowledge, attitudes, perceptions, and practices toward adult vaccination, preventable diseases relevant to adults, and prevention of these diseases. The questions were either single- or multiple-response questions. The latter questions provided multiple-choice answers, or answers on a five-point Likert or rank order scale. The survey also included a few open-ended questions to assess the main reasons for accepting, refusing, recommending, or not recommending specific vaccines.

### Data analysis

Descriptive analysis used counts and percentages, or means and standard deviations as appropriate. For the secondary objective, we stratified data from the general population and HCPs by various sociodemographic variables for statistical analysis. We used a Pearson’s χ^2^ test, or a Fisher’s exact test when expected cell counts were below the threshold for χ^2^ assumptions, to determine whether categorical data were statistically different between subgroups. A p-value <0.05 indicated a significant difference. All statistical analyses were performed using Stata versions 15 and 19 (StataCorp LLC, College Station, TX, USA).

## Results

### Participants and demographic data

A total of 1,169 adults from the general population participated in the study, of whom 64% were women (n = 748). The mean age was 49.8 years (standard deviation [SD] 15.8), and the largest group consisted of the 41–60 year-olds (41.8%), followed by the 18–40 years-olds (30.1%). Of these, 37.9% were unemployed, and most had either completed middle school or high school. Participants reported specific comorbidities: hypertension (24.5%), diabetes (18.8%), and obesity (10.2%), while almost half reported no comorbidities. The majority of the health services, were affiliated with either the public health services (Secretaría de Salud) or the social security system (Instituto Mexicano del Seguro Social, IMSS). Among the participants, the large majority had been vaccinated as adults. Most of them resided in either Mexico State (56%) or Mexico City (27.9%) (Table 1).

**Table 1:** Sociodemographic characteristics of the adults from the general population.

Variable		n	%^a^ or mean (SD)
**Total number**		*1,169*	
**Age, in years**		1,168	49.8 (15.8)
	18–40	351	30.1
	41–60	488	41.8
	>60	329	28.2
**Number of inhabitants in each dwelling**		*1,167*	
	1	52	4.5
	2	169	14.5
	3	209	17.9
	4	301	25.8
	5	216	18.5
	>5	220	18.9
**Sex**		*1,169*	
	Male	421	36.0
	Female	748	64.0
**Primary occupation**		*1,167*	
	Employed	325	27.8
	Laborer	63	5.4
	Day laborer	37	3.2
	Self-employed	248	21.3
	Other	494	42.3
**Current work situation**		*1,167*	
	Employed	383	32.8
	Independent	342	29.3
	Unemployed	442	37.9
**Education level**		*1,167*	
	None	19	1.6
	Primary, not completed	84	7.2
	Primary, completed	190	16.3
	Middle school, completed	436	37.4
	High school, completed	352	30.2
	University, completed	86	7.4
**Religion**		*1,167*	
	None	75	6.4
	Catholic	748	64.1
	Protestant/evangelical	97	8.3
	Believer^b^	228	19.5
	Other	19	1.6
**Monthly family income**^**c**^ **(pesos)**		*1,167*	
	None	115	9.9
	<6,000	294	25.2
	6,000–9,999	245	21.0
	10,000–13,999	278	23.8
	≥14,000	95	8.1
	No response	72	6.2
	Don’t know	68	5.8
**Healthcare access in:** ^**#**^		*1,169*	
	No health service	58	5.0
	SSA	628	53.7
	IMSS	494	42.3
	ISSSTE	13	1.1
	Private practice	20	1.7
	SEDENA/Marina	4	0.3
	Other	16	1.4
**Main medical care unit used**		*1,167*	
	Hospital	222	19.0
	IMSS	338	29.0
	Health center	364	31.2
	Medical consult adjacent to pharmacy	149	12.8
	Private practice	87	7.5
	Other	7	0.6
**Has been vaccinated as an adult**		*1,167*	
	No	73	6.3
	Yes	1,094	93.7
**Place where the vaccine was administered**		*1,094*	
	Health center	1,018	93.1
	Private practice	14	1.3
	Medical consult adjacent to pharmacy	5	0.5
	Workplace	16	1.5
	Home	10	0.9
	Other	31	2.8
**Has a medical diagnosis of**:		*1,169*	
	No diagnosis	567	48.5
	Diabetes	220	18.8
	Obesity	119	10.2
	Hypertension	286	24.5
	Cardiovascular disease	29	2.5
	Chronic lung disease	42	3.6
	Immunological disease	41	3.5
	Cancer	29	2.5
	Other disease	28	2.4
**Place of residence**		*1,168*	
	Mexico City	326	27.9
	Mexico State	658	56.3
	Morelos	184	15.8

In italics, the number of participants who were asked the question.

^a^Not all variables sum to 100% due to missing data. ^b^Translated from ‘Creyente’, a common term in Mexico to refer to people with a nonspecific type of religious preference. ^c^monthly income by household, according to the standard categories used in the national health survey in Mexico (ENSANUT) [35].

# Each option is dichotomous (yes/no), only the yes option is reported.

IMSS, Instituto Mexicano del Seguro Social; n, number; SD, standard deviation; SEDENA, Secretaría de la Defensa Nacional; SSA, Secretaría de Salud.

A total of 228 HCPs participated in the study, with a mean age of 36.5 years (SD 12.2) and of whom 75.9% were women (n = 173). The majority were nurses (n = 137; 60.1%), or general practitioners (n = 64; 28.1%), while few were medical residents (n = 18; 7.9%), or medical specialists (n = 9; 3.9%). All worked at a public institution (data not shown), mostly in primary care. The largest group had worked for ≤5 years in practice, followed by those who had worked for ≥20 years in practice (Table 2).

**Table 2:** Sociodemographic characteristics of the HCPs.

		n	%^a^ or mean (SD)
**Total number**		*228*	
**Age, in years**		225	36.5 (12.2)
	18–40	152	67.6
	>40	73	32.4
**Sex**		228	
	Male	55	24.1
	Female	173	75.9
**Healthcare Profession**		*228*	
	General practitioner	64	28.1
	Medical resident	18	7.9
	Medical specialist	9	3.9
	Nurse	137	60.1
**Years in practice**		*228*	
	≤5	95	41.7
	6–10	35	15.4
	11–15	29	12.7
	16–20	26	11.4
	>20	43	18.9
**Type of medical unit**		*228*	
	Primary care	187	82.0
	Secondary care	41	18.0
**State where you reside**		*228*	
	Mexico City	39	17.1
	Mexico State	71	31.1
	Morelos	118	51.8

In italics, the number of participants who were asked the question.

^a^Not all variables sum to 100% due to missing data.

HCP, healthcare professional; n, number; SD, standard deviation.

### Knowledge of vaccine-preventable diseases and vaccines in the general population

**Fig 1:**
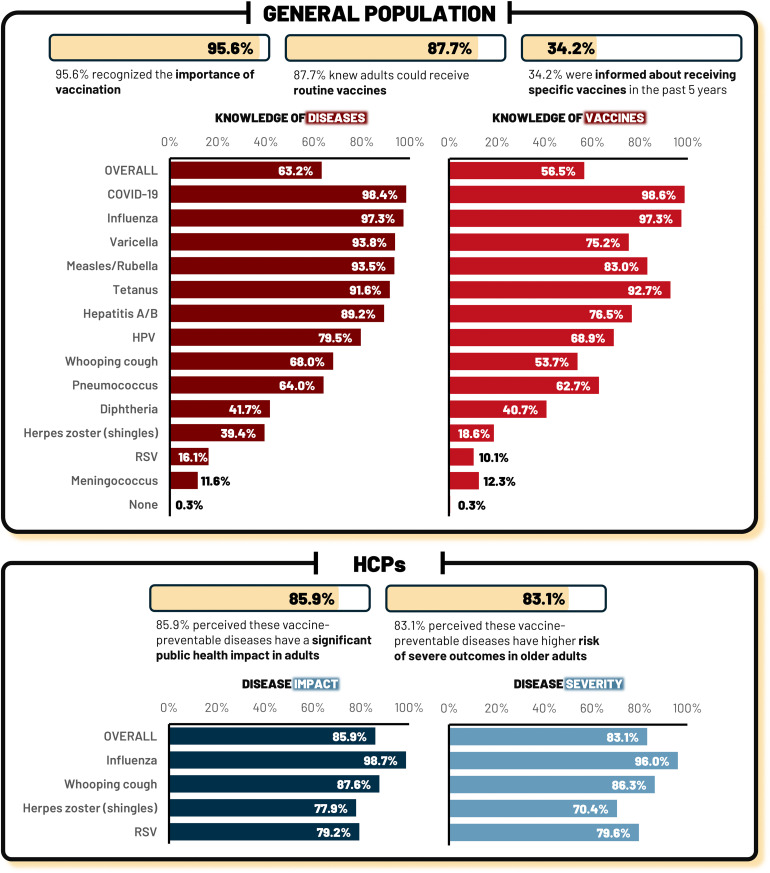
General knowledge of infectious diseases and vaccines among the general population and of disease impact and severity among the HCPs. COVID-19, coronavirus disease 2019; HCP, healthcare professional; HPV, human papillomavirus; RSV, respiratory syncytial virus.

Among the general population, almost all (95.6%) recognized the importance of vaccines, and most (87.7%) knew that adults could receive routine vaccines yet only 34.2% were informed about receiving specific vaccines in the last 5 years. As for knowledge of vaccine-preventable diseases, 68.0% reported having some knowledge about whooping cough, and 39.4% about herpes zoster (HZ), while few had knowledge about RSV. This limited knowledge about RSV was consistent with the knowledge they had about the vaccine; when presented with a list of 13 vaccines, the RSV vaccine, which is not included in the NIP, was the least well-known. To assess the accuracy of these results, we used COVID-19 as a control, which nearly every participant was familiar with (Fig 1).

Fewer adults knew about the infectious nature of whooping cough (33.9%), HZ (26%), and RSV (12.1%) than they did about influenza (77.6%). When asked about the vaccines against these four diseases, most had heard of the influenza vaccine, which is included in the NIP for individuals aged ≥60 years. Fewer had heard of the vaccines against whooping cough (36.3%), HZ (11.1%), or RSV (5.8%), which are not included in the NIP. Of those who knew about the whooping cough, HZ, influenza, and RSV vaccines, 59.7% (253/424), 23.3% (30/129), 58.9% (631/1,072), and 0% (0/68), respectively, learned about them through a health center. Meanwhile, 12.5% (53/424), 34.9% (45/129), 20.5% (220/1,072), and 33.8% (23/68), respectively, learned about them through a vaccination campaign or the media (radio or television). The large majority knew that adults can be vaccinated against influenza and where to obtain the vaccine. In contrast, for HZ, for which the vaccine is not included in the NIP, very few knew that adults can be vaccinated and where to obtain the vaccine (Supplementary S1 Table).

### Perceptions of vaccine-preventable diseases and vaccines in the general population

More than half of the adults believed they could contract the disease if they did not get vaccinated (55.4% for HZ, 63% for RSV, and 82.8% for influenza). These proportions were similar for the perceived risk that if they would not get vaccinated, their family members could also get sick. Furthermore, most participants were aware that vaccines could prevent complications while only a minority viewed vaccination as potentially harmful.

The HZ vaccine is not available in the NIP, and almost half (48.9%) found it too complicated to receive. In contrast, the influenza vaccine is in the NIP, and the majority responded that it is accessible. Most agreed they would get vaccinated if a health professional recommended it (91.7% for HZ, 90.3% for RSV, and 95.6% for influenza).

The majority of respondents were unaware of the safety profile of the HZ and RSV vaccines but knew the influenza vaccine is safe. If others were vaccinated against HZ or RSV, more than half would also get vaccinated and would also get the vaccine if offered in the NIP. For influenza, the majority would get vaccinated if others did, and would accept it if offered in the NIP. On average, 67.8% of adults expressed a positive attitude toward receiving vaccines (Supplementary S2 Table).

The most important factor reported in deciding to get vaccinated or not against HZ, RSV, or influenza was to avoid infection, the second factor was to prevent long-term complications, and the third factor was having information about vaccine safety. For influenza, the most important and second most important factors were the same as for HZ and RSV: to avoid infection, and to prevent complications, while the third factor was convenience rather than information about vaccine safety (Supplementary S1 Fig).

### Attitudes and practices toward seasonal vaccines in the general population

During the winter season before the survey, 71.3% of respondents received the influenza vaccine, while only 29.7% received the pneumococcal vaccine. Healthcare personnel primarily recommended vaccination, with 57% for influenza and 78.4% for pneumococcus. Most vaccinated individuals obtained their vaccines at the public health centres and units (62.9% (735/1,168) for influenza, 94.8% (330/348) for pneumococcus). Almost all respondents felt more confident about getting an influenza or pneumococcal vaccination when recommended by a healthcare professional.

Among those not vaccinated against influenza last winter, the primary reason, for 23.9% (78/326) of unvaccinated individuals, was unavailability of the vaccine (despite being eligible to receive it). An unspecified reason accounted for 27.9% (91/326), while 14.7% (48/326) did not consider the disease severe. For those not vaccinated against pneumococcus last winter, 69.9% (508/727) were not eligible due to age, 21.9% (159/726) cited adverse effects, and 11.3% (82/725) did not consider the disease severe.

### Practices regarding new vaccines among the general population

Regarding new vaccination practices, more than half of respondents stated that they were not very likely or unlikely to get a new vaccine. Respondents stated in similar proportions that, when offered, they would not be very likely or would be unlikely to accept an RSV vaccine (43.5% and 12.2%, respectively), or an HZ vaccine (42.6% and 12.5%, respectively). Overall, almost half of respondents stated that to accept a new vaccine, they need information about the disease it prevents. Of all respondents, 57.7% reported having a vaccination card, but few presented it at the time of the survey (Supplementary S3 Table).

### Knowledge and perception of diseases and vaccines among HCPs

Most HCPs had never treated a case of whooping cough (91.2%). Additionally, 54.9% had not been vaccinated against it in the last five years, even though this vaccination was available at the workplace of most of the HCPs. While 75.2% said they routinely recommend it, only 52.2% specifically recommended the whooping cough vaccine to older adults. Only 45.6% of HCPs knew about the recommendation to vaccinate both pregnant women and adult men and women against whooping cough. A large majority agreed that whooping cough has a significant impact on adults, and that older adults face a higher risk of severity (Supplementary S4 Table and Fig 1).

More than half of the HCPs had treated influenza cases, and almost all had received the vaccination in the last five years. Furthermore, almost all stated that they routinely recommend influenza vaccination to adults, and reported recommending it to older adults. Most (91.6%) recommend that at-risk groups take one dose each winter, and almost all agreed that influenza has a significant impact on adults and that there is a higher risk of severe illness in older adults (Supplementary S4 Table and Fig 1).

The majority of HCPs (63.7%) had never treated an HZ case. Most HCPs stated that an HZ vaccination service was not available at their workplace, and had not received an HZ vaccination themselves in the last five years. Most HCPs (75.2%) had not recommended the vaccine to older adults, and 62.8% do not usually recom mend it. Moreover, more than half did not recall the vaccination schedule. Most HCPs (77.9%) agreed that HZ has a significant impact on adults, and 70.4% agreed that there is a higher risk of severity in older adults (Supplementary S4 Table and Fig 1).

The majority of HCPs had never treated a case of RSV, nevertheless, they agreed that RSV has a high disease burden (Supplementary S4 Table), that RSV has a high impact on adults, and that older adults are at higher risk of severe outcomes (Fig 1).

Overall, 85.9% of HCPs perceived that the vaccine-preventable diseases whooping cough, influenza, HZ, and RSV have a significant public health impact in adults. Additionally, 83.1% perceived that these vaccine-preventable diseases have a high risk of severe outcomes in older adults (Fig 1).

### Perceptions of vaccine-preventable diseases and vaccines among HCPs

The three main factors for HCPs to accept and recommend vaccination against HZ, RSV, influenza, and whooping cough ranked the same for these diseases. The main factors were knowledge of the disease, knowledge of complications, and knowledge of efficacy and effectiveness (Supplementary S1 Fig).

### Vaccination KAP among the general population by sociodemographic variables

To determine whether, among the general population, any of the sociodemographic variables — sex, age group (≤40 years, 41–60 years, or >60 years), occupation, or education — were associated with KAP regarding vaccines, we analyzed the data after stratifying by these variables.

#### Stratified by sex.

Stratification by sex showed that most knowledge of the HZ, influenza, and RSV vaccines did not differ significantly between men and women. However, a difference was found in the proportion of women and men who disagreed that the HZ vaccine might be harmful (48.9% (365/747) in women, 43.0% (180/421) in men, p = 0.002), and a small difference in the proportion of people who had heard about the influenza vaccine (92.9% (694/747) in women, 90.2% (378/421) in men, p = 0.003).

#### Stratified by age.

Stratification by age revealed that, regarding HZ, while the majority of participants did not have knowledge of the disease, that lack was greatest in the oldest participants at 65.5% (215/329) in >60-year-olds, compared to 59.0% (207/351) in ≤40-year-olds and 60.0% (292/488) in 41–60-year-olds (p < 0.001). As for the statement that vaccination would protect them, 80.6% (283/351) of the ≤ 40-year-olds, 78.4% (381/488) of the 41–60-year-olds, and 76.2% (250/329) of the > 60-year-olds agreed (p = 0.002). Regarding influenza, many outcomes differed significantly between age groups, with the youngest group generally having more knowledge. For instance, 85.5% (300/351) of the ≤ 40-year-olds, 79.3% (386/488) of the 41–60-year-olds, and 66.8% (219/329) of the > 60-year-olds knew that influenza is caused by a virus (p < 0.001). Similarly, for RSV, some outcomes differed significantly between age groups, with the youngest group having more knowledge. For instance, 81.8% (287/351) of the ≤ 40-year-olds, 79.8% (388/488) of the 41–60-year-olds, and 74.1% (243/329) of the > 60-year-olds knew that vaccination would protect them (p < 0.01).

#### Stratified by occupation.

Stratification by occupation showed that, regarding HZ, the majority did not know the disease, with 50.9% (165/325) of employees, 68% (68/100) of laborers, 59.3% (147/248) of employers and self-employed, and 67.5% (333/494) of other occupations not knowing the cause of HZ. The difference between these groups was statistically significant (p < 0.001). In contrast, regarding influenza, the majority knew it is caused by a virus, although there was a statistically significant difference in knowledge among occupational groups (p < 0.001), with laborers being the least aware. Similar to HZ, the majority did not know about RSV, with a statistically significant difference in knowledge among occupational groups (p = 0.009), again with laborers being least aware of the cause.

#### Stratified by education.

Stratification by education revealed significant differences in the KAP of HZ among education groups. The largest proportion of people who had no knowledge of the cause of HZ was among those who did not complete primary school (75.5%, 77/103), followed by those who completed primary school (72.6%, 138/190), middle school (65.8%, 287/436), high school (52.0%, 183/352), or university (32.9%, 28/86) (p < 0.001). Similar differences in knowledge were observed between education groups when analyzing KAP of influenza and RSV.

#### Following recommendations.

When asked whose recommendation they would follow for vaccination, no significant differences for HZ, influenza, or RSV were observed between men and women, age groups, or education levels. When asked whose recommendation they would follow for vaccination, the only significant difference found was between occupation groups for influenza. Laborers were the least likely to get vaccinated against influenza based on a family member’s or partner’s recommendation (71.0%, 71/100). They were followed by individuals in other occupations (73.0%, 359/494), employees (81.5%, 265/325), and employers (82.2%, 203/248) (p = 0.003).

### Vaccination KAP among the HCPs by sociodemographic variables

To determine whether sociodemographic variables among HCPs — sex, age group (≤40 years, > 40 years), profession, or years in practice (≤5, 6–10, 11–15, 16–20, or >20 years) — affected KAP of the vaccines against HZ, influenza, and RSV, we analyzed the data after stratification by these variables.

#### Stratified by sex.

Stratification by sex showed that fewer women (29.2%, 50/173) than men (58.2%, 32/55) had ever treated an HZ case (p < 0.001), and that fewer women (17.6%, 30/173) than men (36.4%, 20/55) had ever treated an RSV case (p = 0.012). Most HCPs had treated influenza cases, although more men (69.1%, 38/55) than women (51.5%, 88/173) had done so (p = 0.04). Stratification by sex, age, or profession revealed that more female (95.3%, 163/173) than male (78.2%, 43/55) HCPs had never treated a whooping cough case (p < 0.001), and that more nurses (96.3%, 131/137) than physicians (83.3%, 75/91) had never treated a whooping cough case (p < 0.001).

#### Stratified by age.

Stratification by age group showed that most KAP about HZ, influenza, and RSV did not differ between age groups. However, more younger HCPs agreed with the statement “HZ has an impact on population health”, than older HCPs (p = 0.021).

#### Stratified by profession.

Stratification by profession showed that 38.9% (35/91) of physicians and 80.1% (109/137) of nurses reported never having treated HZ cases (p < 0.001). Additionally, 66.3% (59/91) of physicians and 82.4% (112/137) of nurses stated they had never treated RSV cases (p = 0.014). When asked if “HZ has an impact on population health”, 31.1% (28/91) of physicians and 23.5% (32/137) of nurses disagreed (p = 0.017). The same question about RSV revealed that 16.7% (15/91) of physicians and 24.3% (33/137) of nurses disagreed (p = 0.009).

#### Stratified by years in practice.

Stratification by years in practice allowed examining whether this factor affected perceptions about the impact of HZ on population health. We found a significant difference in agreement with the statement “HZ has an impact on population health”. HCPs with the fewest years in practice (1–5 years) showed 80.6% (75/95) agreement, followed by those in practice for 6–10 years (80.0%, 28/35), 11–15 years (48.3%, 14/29), 16–20 years (53.8%, 14/26), and those with >20 years in practice (48.8%, 21/43) (p < 0.001). Furthermore, none of the groups generally recommended the vaccine, which is understandable given that it is not included in the NIP and is not widely available.

## Discussion

We conducted an observational cross-sectional study to describe the KAP concerning adult vaccination among adults and HCPs in Mexico. Overall, the general population demonstrated knowledge about the importance of vaccination (95.6%). Although unfamiliar with some diseases and their causes, the general population perceived that vaccination could prevent infections and their complications. The general population trusted vaccination recommendations most when made by an HCP. HCPs were generally knowledgeable about the diseases and the recommended vaccination schedules for adults. They indicated that before recommending a new vaccine, they would need to learn more about the disease it prevents.

The vast majority of the general population had a good overall knowledge of the role of vaccines in preventing infectious diseases. They were mostly in favor of receiving vaccines, and 91.7% would get vaccinated for HZ, 90.3% for RSV, and 95.6% for influenza if an HCP recommended it. Such positive attitudes of Mexican adults toward vaccination were also found in a KAP study about dengue vaccination that included 600 adults [17] and in a study about perspectives about maternal immunization among pregnant women [18]. Despite the overwhelmingly positive attitude toward vaccination, only 29.7% reported having received the pneumococcal vaccine, and 71.3% reported having received the influenza vaccine before the last winter season. These vaccination proportions are similar to those reported in a study from 2022, which highlighted that among Mexican adults >60 years old, only 24.4% received the pneumococcal and 49.1% the influenza vaccine at the start of the previous winter season [8]. They are also similar to a study in Spain where among adults ≥65 years, 29% received the pneumococcal and 80% the influenza vaccine [19]. Among our respondents, the main factors for not getting vaccinated were, for the pneumococcal vaccine, not being eligible based on age, and for the influenza vaccine, being eligible but the vaccine was not available where they lived.

Despite an overall knowledge of vaccines and the support of vaccination in the general population, we identified substantial knowledge gaps regarding vaccine-preventable diseases and their complications. For instance, while 98.4% knew about COVID-19, and 98.6% knew a vaccine exists against COVID-19, only 39.4% knew about HZ, and only 18.6% knew a vaccine exists against HZ. In other studies also a large knowledge gap was found between the diseases, for example a study in Greece reported that 98.2% had sufficient knowledge about the COVID-19 vaccine, while only 56% had sufficient knowledge about the pneumococcal vaccine [20], In contrast, in a survey among over 8,000 adults conducted in Saudi Arabia, 64% had heard of HZ, and 58.8% had heard of an HZ vaccine [21]. Those proportions were much higher than the 39.4% and 18.6% in our participants. The difference may be partly explained by a vast difference in education level, with 98.0% of the Saudi Arabian participants having secondary or tertiary education [21], while in our participants this was only 37.5%. Another explanation for the difference is that in Saudi Arabia, vaccines were available and promoted during intensive national communication campaigns. As we found large differences in awareness of HZ and the existence of an HZ vaccine between education levels, this may explain some of the lower HZ knowledge in our participants. Another difference, although not directly comparable as ages were categorized differently, is that the participants in the Saudi Arabian study [21] were younger than those in our study (72.8% < 50-year-olds in Saudi Arabia, vs. 30.1% ≤ 40-year-olds and 41.8% 41–60-year-olds in our study). This age difference may be relevant because we found that younger participants had significantly more knowledge of HZ than older participants. Similarly, younger participants also had more knowledge of influenza, but this was not the case for RSV.

In our general population, awareness that whooping cough, HZ, and RSV diseases have infectious causes was low (12.1–33.9%), and awareness that vaccines are available against these infections, specifically targeting adults, was also low (5.8–36.3%). These findings align with a systematic review that found limited knowledge about RSV vaccination among adults [22]. For instance, a study in the United States among 827 adults at risk for RSV reported that only 43.3% of respondents had heard of RSV [23], a proportion that is almost four times higher than in our study (12.1%). The difference could be related to higher health literacy, or the promotion and availability of the RSV vaccine for adults. In general, women are more likely to accept vaccines than men. In Mexico, women traditionally visit medical units more frequently than men; and women are also responsible for the health of children and the family in general. The general population was most likely to get vaccinated if an HCP recommends a vaccine. This indicates a general trust in the advice of HCPs, which was also noted in other studies as a review of studies worldwide, including Mexico, on attitudes toward influenza vaccine uptake among adults found that trust in healthcare workers was the most important promoter of vaccine uptake [24]. Similarly, in pregnant women in five Latin American countries, including Mexico, trust in HCP recommendations was an important driver of vaccination [18]. Thus, both globally and in Mexico, the KAP of HCPs regarding adult vaccination plays a crucial role in achieving good vaccine coverage. A literature review found that worldwide, HCPs are the most trusted source for vaccination advice [25]. Another important factor in getting vaccinated was seeing others in their age group getting vaccinated. Although respondents were unaware of the vaccines’ efficacy, they felt confident if others had already received the vaccines. The primary motivation for vaccination is to avoid infections and, secondarily, to avoid complications. Most participants report attending primary care units for vaccinations, yet 12.3% are unaware of vaccines specifically targeted at adults.

As trust in HCP recommendations has been reported as an important driver of vaccination, it may be worth noting that participants in many studies report a lack of counselling on adult vaccinations by HCPs. For instance, in a KAP study in India, only 62% of adults eligible for adult vaccination were recommended adult vaccinations [26], while in Turkey, only 59% of adults were recommended adult vaccinations [27]. Also in our study, not all HCPs recommended all relevant adult vaccinations as they lacked the necessary knowledge. Hence, improvement of HCPs’ knowledge may improve recommendations and consequently vaccination rates.

The behavior of the respondents in this study, who, although they were mostly in favor of vaccination, mainly received vaccination for diseases they considered themselves susceptible to or they considered severe, e.g., influenza, is in line with the HBM. The HBM states that people are likely to engage in disease-prevention behaviors, such as vaccination, if they perceive they are susceptible to the disease, the disease is severe, and barriers are minimal [15].

We found that for influenza, age was associated with willingness to get vaccinated if a vaccine was offered. Individuals aged 18–40 or 41–60 years were more likely to get vaccinated than those over 60. For HZ and RSV, the willingness to get vaccinated was not associated with age. In a study of 1,512 Mexicans about willingness to get vaccinated against COVID-19 before the vaccine rollout, younger groups (18–24 and 25–34 years) were more likely to be willing to get vaccinated [28]. However, the data cannot be compared directly as the age groups differ.

Among HCPs, overall knowledge about adult vaccinations was good, although correct recollection of the vaccination schedules for some vaccines, namely whooping cough and HZ, was limited. Similarly, a report from the United States, where the RSV vaccine is available for adults, showed that HCPs were not fully aware of RSV vaccine recommendations [29]. In our study, important factors for HCPs to accept or recommend a vaccine included knowledge of the disease, its complications, and the vaccine’s efficacy and effectiveness. This is in line with a systematic review of studies on primary care physician vaccination behavior, which indicated that a lack of knowledge among these providers hindered making recommendations to patients [30]. Similarly, a KAP study among HCPs in Honduras about influenza vaccination found a significant association between vaccine knowledge score and attitude score [31]. Other studies have shown that HCPs expressed a desire for more knowledge of vaccine-preventable diseases and vaccines. For instance, a systematic review about KAP of RSV found that HCPs often wanted more knowledge of the disease [22]. A report about KAP of RSV among HCPs in Jordan indicated that most had low awareness of RSV or RSV vaccines and needed more information on RSV vaccines [32]. At the time of this study, in 2024, RSV vaccines were not yet available in Mexico for adults. Therefore, we did not assess the HCPs’ specific knowledge of RSV and recommendations. However, educating HCPs about RSV and RSV vaccination before rollout may improve vaccine recommendations and uptake. Education is especially important, as misconceptions about vaccination-associated adverse events can greatly reduce vaccination rates. This was reported for influenza vaccination coverage among 947 HCPs, where vaccination coverage of HCPs was halved in 2018 compared to 2017 due to misconceptions [31]. Although almost half of the HCPs had over five years of work experience, very few reported ever treating patients with whooping cough, RSV, or HZ. There were more nurses than medical doctors among the HCPs, which explains why most had never treated patients with these diseases. Nonetheless, the HCPs favored vaccinations against these diseases and recommended that the population follow age-specific recommendations.

This study has several limitations. Firstly, it used surveys, which carry the risk of classification bias if questions are suggestive and of interviewer bias. To minimize this risk, we only hired interviewers trained in conducting surveys. Secondly, the higher number of nurses compared to medical doctors among HCPs could have biased the outcomes. This is evident in the high proportions that had never treated a whooping cough, HZ, or RSV case, as treatment is provided by medical doctors. Therefore, caution is advised when interpreting the results about disease burden and treatment. However, the findings about the vaccines and vaccination program are particularly relevant as in Mexico, nurses generally manage the vaccination program. Thirdly, when approaching people to participate in surveys, there is a risk of selection bias. To reduce this risk, we calculated in advance adequate sample sizes for both participant groups. Fourthly, since sample size was based on the feasibility of recruitment, the age distribution (i.e., the proportion of the population aged 50–59 years or ≥60 years) in the study sample may not reflect the actual age distribution in Mexico. Fifthly, as a self-reported study, recall bias cannot be excluded, nor are the responses verified, so social desirability bias cannot be excluded either. As the vaccination cards were not available, we were unable to assess the actual vaccine coverage or validate utilization responses by comparing them with the data on the cards. Hence, vaccination rates should be interpreted with caution, as studies have shown that these biases can affect the outcome in both direction and magnitude [33,34]. Sixthly, although we had sufficient power from the large sample size, and one of the strengths of the study was sample representativeness, we could not perform multivariable logistic regression to adjust for confounding due to the structure of the data. Lastly, as the study involved Mexico City and only two of the 31 Mexican states, the results cannot be generalized to Mexico as a whole.

## Conclusion

Our results underscore the need to strengthen health education and communication strategies aimed at the adult population, and to address barriers that hinder vaccination access, particularly for less well-known vaccines. Improving knowledge among adults may increase vaccine acceptance and coverage. Additionally, providing HCPs with more knowledge of vaccines and vaccination recommendations could strengthen their awareness to recommend these vaccines. Our findings are important for supporting adult disease awareness initiatives and decision-making in Mexico and other Latin American countries. Efforts to provide tailored information on vaccine-preventable adult diseases may help HCPs promote immunization, thereby reducing vaccine-preventable diseases and enhancing public health.

## Supporting information

S1 Appendix Sample calculation and sampling procedure. (DOCX)

S1 Table Knowledge about whooping cough, herpes zoster, influenza, and RSV in the general population. (DOCX)

S2 Table Perceptions about herpes zoster, RSV, and influenza (illness and vaccine) in the general population. (DOCX)

S3 Table Practices regarding new vaccines among the general population. (DOCX)

S4 Table Knowledge and perceptions among HCPs (N = 228). (DOCX)

S1 Fig Factors in deciding to vaccinate among the general population and for accepting or recommending a vaccine among HCPs. (TIF)

## Abbreviations

COVID-19: coronavirus disease 2019
HBM: Health Belief Model
HCP: healthcare professional
HZ: herpes zoster
KAP: knowledge, attitudes, and practices
NIP: national immunization program
RSV: respiratory syncytial virus
SD: standard deviation
SNM: Social Norms Model
WHO: World Health Organization
