# Supplementary material for: Knowledge, attitudes, and practices related to adult vaccination among adults and healthcare professionals across Mexico
Source: PLoS One. 2026 May 21;21(5):e0348625. doi: 10.1371/journal.pone.0348625 (PMC13193386; doi:10.1371/journal.pone.0348625)
Supplement: S1 Appendix — (DOCX) [file pone.0348625.s001.docx]

## **S1 Appendix. Sample calculation and sampling procedure**

Considering the recruitment feasibility and acceptable margin of error, the sample size for the study population was estimated at approximately 1,200 adults of either sex, aged ≥20 years, who attended primary healthcare units affiliated with the social security institutions (IMSS, ISSSTE, or the Ministry of Health), and 200 healthcare professionals (HCPs). The sample calculation details are described in this section.

## **Target Population**

The targeted healthcare units in the study represent the first level of healthcare, which is the closest and most accessible to the general population. These units provide care and services nationwide through a network of medical units offering essential services, depending on their infrastructure and capacity. Although there may be variations in the type and scope of services provided, the following are some of the most common services offered: family medicine consultations, preventive medicine, dental care, social work, continuous medical care, or emergency services (in some cases), nutrition, psychology, epidemiology, clinical laboratory, imaging, and pharmacy. Some medical units are linked to hospitals or other specialized medical care units, such as Addiction Primary Care Centers or Medical Specialty Units, which offer specialized care for chronic diseases.

The sample size of a survey depends on the parameters to be estimated, the variability of the attribute of interest in the population, the sampling procedure, and the precision required for the estimates. The parameter of interest in this proposal was the prevalence of knowledge and acceptance of vaccination among adults in Mexico. Based on the National Health and Nutrition Survey (ENSANUT) in Mexico, among 6,547 adults aged 20–59 years and 2,048 adults aged ≥60 years conducted in 2020, we initially assumed the prevalence of vaccination acceptance and coverage was around 50% [1]. This assumption was based on responses to both influenza and coronavirus disease 2019 (COVID-19) acceptance questions in adults aged ≥60 years. In the survey, 69% (95% confidence interval [CI]: 66.3%–71.5%) reported willingness to receive the influenza vaccine in the next season (October–December 2020), and 53.9% (95% CI: 50.9%–56.9%) expressed willingness to accept the COVID-19 vaccine when available [1].

## **Sampling Procedure**

The study followed a cluster sampling approach for the adult population, and convenience sampling for the HCP population. Public documents exist for each state that list the number of healthcare units. For example, the state of Morelos, which is divided into 33 municipalities, has 274 healthcare units of the first level of care (224 Health Centers, 24 IMSS Family Medicine Units, and 26 ISSSTE Family Medicine Units).

For adult recruitment, the primary sampling procedure occurred in two stages: First, all the primary healthcare units within each state and municipality were clustered, and then the final number of healthcare units was selected based on the number of medical offices within each unit. So, healthcare units were selected by probabilistic sampling, with the probability proportional to the number of medical offices in each unit. Then, in the second stage, a defined number of participants were interviewed outside the selected healthcare units. In addition, to avoid bias, the sample of participants was distributed throughout the unit’s opening hours. For HCP recruitment, convenience sampling was applied, with the primary sampling at society or academic meeting centers where HCPs meet outside of their medical units.

## **Sample Calculation**

The formula used to estimate the sample size of 1,200 adult participants was:


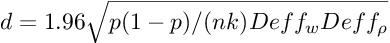


Based on a design effect (Deff_w_= 1.5) and (Deff_ρ_= 1.62), we expected a confidence interval (CI) half-width of 4.7% if the sample consisted of 45 clusters (n) with 25 interviews (k) each; Deff_w_= 1.5. Deff_w_ = 1 + CV^2^(w) can approximate the design effect due to variability of the weights [2]; Deff_w_ was estimated based on the National Survey on Health and Nutrition (ENSANUT) 2020 of Mexican adults ≥20 years old [1, 3]. Similarly, Deff_ρ_ = 1 + ρ(k-1) can approximate the design effect due to the homogeneity of clusters; ρ (ρ=0.026) was estimated based on the average of the intra-cluster correlation of six variables that measured acceptance of vaccination or vaccination in the ENSANUT 2020 [1, 3].

To estimate the sample size of 200 HCPs, we assumed that quota sampling would be similar to simple random sampling (SRS). Then Deff=1, and a sample size of n=190 would provide confidence intervals with a sem-amplitude of d=7.1% for a prevalence of 50%. We assumed SRS as an approximation to compute a sample size, but we are aware that it is not straightforward to compute confidence intervals for quota sampling.

## **Cluster sampling**

## **Target population**

Healthcare users of first-level medical units in the states of Mexico City, México, and Morelos that belong to IMSS (Mexican Institute for Social Security ) or SSa (Minister of Health). IMSS is the main provider of medical services for people with formal employment in the private sector, and SSa is the main provider for people without formal employment. According to the ENSANUT 2024 (National Health Survey of Mexico), healthcare users attended medical units as follows: IMSS (25%), SSa (16%), Private services (49%), ISSSTE-PEMEX-SEDENA (9%); where ISSSTE-PEMEX-SEDENA are the providers of medical services for government employees.

## **Sampling procedure**

The sampling frame included medical units of IMSS (150) and medical units of SSa (1,343). From these, we probabilistically selected 22 units from IMSS and 23 units from SSa; selection was made with systematic sampling and probability proportional to the number of medical offices. Next, an exit sample of 25 healthcare users was obtained from each medical unit. Therefore, the largest medical units had a greater probability of selection, and the expected sampling size was about 1,125.

## **Sampling size**

Sample size was estimated to obtain 95% confidence intervals with a semi-amplitude close to d=5% for a prevalence of 63%. The factors used to estimate the sample size were:

- A prevalence of p=63% of people willing to accept a COVID-vaccine, p was estimated from ENSANUT 2020 (adults 20-59 years old); p was chosen as a prevalence that could be of interest for this survey.
- n= 45 medical units to be visited, and k= 25 healthcare users to be interviewed per medical unit
- Deff_ρ_ = 1 + ρ(k-1) = 1.72, is the design effect due to the intra-cluster correlation coefficient. ρ = 0.03 was estimated based on ENSANUT 2020.
- Deff_w_ = 1.40 is the design effect due to the variability of the weights
- We used the following formula to estimate d=4.4%.


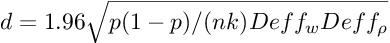


## **Weighting strategy**

We did not apply weighting for three reasons:

1. The probability of selecting a participant was


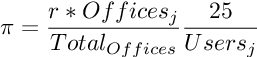


Where r is the number of medical units selected in the stratum, Offices_j_  is the number of medical offices in the j-medical unit, Total_Offices_ is the total number of medical offices in the stratum. Then, if we assume that the number of participants is proportional to the number of medical offices; then, in each stratum, the sampling design is approximately self-weighted.

1. The IMSS strata and SSa strata were allocated a similar sample size, plus the assumption that sampling is self-weighted in each strata, is equivalent to assuming that the sizes of the populations of IMSS and SSa are equal. In contrast, ENSANUT 2020 showed that the population size of IMSS and SSa are similar: a) as 61% of people attended IMSS with a health necessity during the last three months, and 39% attended SSa; and b) 40% of people have the right to be attended by IMSS, whereas 60% of people, do not have health insurance and may be attended by SSa. Unfortunately, we did not have official reports on the number of users of the medical units in the sample; therefore, we assumed that the population sizes of IMSS and SSa are similar and the prevalences of variables about vaccination are similar, as shown next.
2. The below Table presents vaccination prevalences in two subsets. IMSS are adults (20-59 years old) who have the right to attend IMSS, and SSa are adults who do not have health insurance, so they are potential SSa users. The Table shows that the simple average of the prevalences on IMSS and SSa is similar to the prevalences of the total and average of SSa and IMSS. Therefore, giving the same weight to IMSS and SSa should have minor effects on the estimations of this survey.

## **Table**

|  | **IMSS** | **SSa** | **Total** | **Average (IMSS, SSa)** |
| --- | --- | --- | --- | --- |
| Since the last September, ¿ Have you received the influenza vaccine ? | 39% | 33% | 36% | 36% |
| ¿ Would you accept the influenza vaccine in the following months ? | 74% | 63% | 68% | 69% |
| ¿ Would you accept the COVID vaccine ( when it is available) ? | 70% | 58% | 63% | 64% |

## **References**

1. Shamah-Levy T, Romero-Martínez M, Barrientos-Gutiérrez T, Cuevas-Nasu L, Bautista-Arredondo S, Colchero MA, et al. Encuesta nacional de salud y nutrición 2020 sobre Covid-19. Resultados nacionales. Cuernavaca, Mexico: Instituto Nacional de Salud Pública; 2021 [cited July 7, 2025]. Available from: <https://ensanut.insp.mx/encuestas/ensanutcontinua2020/doctos/informes/ensanutCovid19ResultadosNacionales.pdf>.

2. Chatrchi G, Brisebois F. Survey weighting adjustments and the design effect: A case study. Joint Statistical Meetings 2015; Seattle WA: American Statistical Association,; 2015. p. 414-26. Available from: <http://www.asasrms.org/Proceedings/y2015/files/233907.pdf>.

3. Romero-Martínez M, Shamah-Levy T, Franco-Núñez A, Villalpando S, Cuevas-Nasu L, Gutiérrez JP, et al. National Health and Nutrition Survey 2012: design and coverage. Salud Pública de México [Internet]. 2013; 55:[S332-S40 pp.]. Available from: <https://www.scielo.org.mx/scielo.php?script=sci_arttext&pid=S0036-36342013000800033&lng=es&tlng=es>.
