# Supplementary material for: Knowledge, attitudes, and practices related to adult vaccination among adults and healthcare professionals across Mexico
Source: PLoS One. 2026 May 21;21(5):e0348625. doi: 10.1371/journal.pone.0348625 (PMC13193386; doi:10.1371/journal.pone.0348625)
Supplement: S1 Table — (DOCX) [file pone.0348625.s002.docx]

**S1 Table.** **Knowledge about whooping cough, herpes zoster, influenza, and RSV in the general population**

|  |  |  | **Whooping cough (W)** | | **Herpes zoster (HZ)** | | **Influenza (I)** | | **RSV** | |
| --- | --- | --- | --- | --- | --- | --- | --- | --- | --- | --- |
|  |  | **Knowledge, diseases and vaccines** | **n** | **%*** | **n** | **%*** | **n** | **%*** | **n** | **%*** |
|  |  | Total number | *1,169* |  | *1,169* |  | *1,169* |  | *1,169* |  |
| **About the diseases (W, HZ, I, RSV)** | | |  |  |  |  |  |  |  |  |
|  | Do you know what it is? | | *1,167* |  | *1,167* |  | *1,167* |  | *1,167* |  |
|  |  | Chronic disease | 251 | 21.5 | 36 | 3.1 | 121 | 10.4 | 32 | 2.7 |
|  |  | Bacterial (W)/viral (HZ, I, RSV) infection | 396 | 33.9 | 303 | 26.0 | 906 | 77.6 | 141 | 12.1 |
|  |  | Stress/anxiety | 3 | 0.3 | 101 | 8.7 | 9 | 0.8 | 1 | 0.1 |
|  |  | Does not exist | 0 | 0.0 | 9 | 0.8 | 1 | 0.1 | 11 | 0.9 |
|  |  | Don’t know | 515 | 44.1 | 715 | 61.3 | 122 | 10.5 | 982 | 84.1 |
|  |  | Other | 2 | 0.2 | 3 | 0.3 | 8 | 0.7 | 0 | 0.0 |
|  | How did you find out? | | *ND* |  | *444* |  | *ND* |  | *174* |  |
|  |  | Family/acquaintance |  |  | 173 | 39.0 |  |  | 28 | 16.1 |
|  |  | Got sick |  |  | 27 | 6.1 |  |  | 2 | 1.1 |
|  |  | Media |  |  | 236 | 53.2 |  |  | 129 | 74.1 |
|  |  | Don’t know |  |  | 8 | 1.8 |  |  | 15 | 8.6 |
| **About the vaccines (W, HZ, I, RSV)** | | |  |  |  |  |  |  |  |  |
|  | Have you heard of the vaccine against…? | | *1,166* |  | *1,166* |  | *1,167* |  | *1,165* |  |
|  |  | No | 700 | 60.0 | 1.005 | 86.2 | 88 | 7.5 | 1.032 | 88.6 |
|  |  | Yes | 423 | 36.3 | 129 | 11.1 | 1.073 | 91.9 | 68 | 5.8 |
|  |  | Don’t know | 43 | 3.7 | 32 | 2.7 | 6 | 0.5 | 65 | 5.6 |
|  | How did you find out about the vaccine? | | *424* |  | *129* |  | *1.072* |  | *68* |  |
|  |  | Health center | 253 | 59.7 | 30 | 23.3 | 631 | 58.9 | 0 | 0 |
|  |  | Hospital | 72 | 17.0 | 11 | 8.5 | 169 | 15.8 | 0 | 0 |
|  |  | Education (college, university) | 15 | 3.5 | 7 | 5.4 | 30 | 2.8 | 0 | 0.0 |
|  |  | Vaccination campaign | 43 | 10.1 | 6 | 4.7 | 157 | 14.6 | 0 | 0.0 |
|  |  | Television/radio | 10 | 2.4 | 39 | 30.2 | 63 | 5.9 | 23 | 33.8 |
|  |  | Internet | 11 | 2.6 | 19 | 14.7 | 9 | 0.8 | 26 | 38.2 |
|  |  | Family/acquaintance | 11 | 2.6 | 17 | 13.2 | 11 | 1.0 | 11 | 16.2 |
|  |  | Don’t remember | 8 | 1.9 | 0 | 0.0 | 2 | 0.2 | 8 | 11.8 |
|  |  | Don’t know | 1 | 0.2 | 0 | 0.0 | 0 | 0.0 | 0 | 0.0 |
|  | Who can get vaccinated (W, HZ, I)?^#^ | |  |  |  |  |  |  |  |  |
|  |  | Children/adolescents | 383 | 32.8 | 20 | 1.7 | 845 | 72.3 | ND |  |
|  |  | Adults | 196 | 16.8 | 101 | 8.6 | 1.029 | 88 | ND |  |
|  |  | Don’t know | 20 | 1.7 | 21 | 1.8 | 19 | 1.6 | ND |  |
|  | Do you know where to get the vaccine (W, HZ, I)? | | *423* |  | *129* |  | *1.071* |  | *ND* |  |
|  |  | No | 31 | 2.7 | 45 | 3.8 | 52 | 4.4 |  |  |
|  |  | Yes | 390 | 33.4 | 79 | 6.8 | 1.012 | 86.6 |  |  |
|  |  | Don’t know | 2 | 0.2 | 5 | 0.4 | 7 | 0.6 |  | - |

In italics, the number of participants who were asked the question.

*The percentages do not add up to 100% due to missing data. ^#^Each option is dichotomous (yes/no).

n, number; ND, not determined (not asked); RSV. respiratory syncytial virus.
