# Supplementary material for: Knowledge, attitudes, and practices related to adult vaccination among adults and healthcare professionals across Mexico
Source: PLoS One. 2026 May 21;21(5):e0348625. doi: 10.1371/journal.pone.0348625 (PMC13193386; doi:10.1371/journal.pone.0348625)
Supplement: S2 Table — (DOCX) [file pone.0348625.s003.docx]

**S2 Table.** **Perceptions about herpes zoster, RSV, and influenza (illness and vaccine) in the general population**

|  |  |  | **Herpes zoster (HZ)** | | **RSV** | | **Influenza (I)** | |
| --- | --- | --- | --- | --- | --- | --- | --- | --- |
|  |  | **Perceptions about illness and vaccines** | **n** | **%*** | **n** | **%*** | **n** | **%*** |
|  |  | **Total number** | *1,169* |  | *1,169* |  | *1,169* |  |
| **Susceptibility to illness (HZ, RSV, I)** | | |  |  |  |  |  |  |
|  | If I don't get vaccinated, I could get infected | | *1,166* |  | *1,166* |  | *1,166* |  |
|  |  | Do not agree | 271 | 23.2 | 189 | 16.2 | 180 | 15.4 |
|  |  | Agree | 646 | 55.4 | 734 | 63.0 | 966 | 82.8 |
|  |  | Don’t know | 249 | 21.4 | 243 | 20.8 | 20 | 1.7 |
|  | If I don't get vaccinated, my family members may get sick | | *1,166* |  | *1,166* |  | *1,166* |  |
|  |  | Do not agree | 337 | 28.9 | 256 | 22.0 | 250 | 21.4 |
|  |  | Agree | 574 | 49.2 | 679 | 58.2 | 891 | 76.4 |
|  |  | Don’t know | 255 | 21.9 | 231 | 19.8 | 25 | 2.1 |
| **Severity of the disease (HZ, RSV, I)** | | |  |  |  |  |  |  |
|  | The vaccine can prevent complications | | *1,166* |  | *1,166* |  | *1,166* |  |
|  |  | Do not agree | 28 | 2.4 | 23 | 2.0 | 41 | 3.5 |
|  |  | Agree | 879 | 75.4 | 896 | 76.8 | 1,090 | 93.5 |
|  |  | Don’t know | 259 | 22.2 | 247 | 21.2 | 35 | 3.0 |
|  | Sick people are at greater risk of suffering | | *1,166* |  | *1,166* |  | *1,166* |  |
|  |  | Do not agree | 136 | 11.7 | 106 | 9.1 | 80 | 6.9 |
|  |  | Agree | 800 | 68.6 | 838 | 71.9 | 1,041 | 89.3 |
|  |  | Don’t know | 230 | 19.7 | 222 | 19.0 | 45 | 3.9 |
|  | Healthy people can also suffer | | *1,166* |  | *1,166* |  | *1,166* |  |
|  |  | Do not agree | 151 | 13.0 | 111 | 9.5 | 92 | 7.9 |
|  |  | Agree | 884 | 75.8 | 901 | 77.3 | 1,044 | 89.5 |
|  |  | Don’t know | 131 | 11.2 | 154 | 13.2 | 30 | 2.6 |
| **Vaccine benefits (HZ, RSV, I)** | | |  |  |  |  |  |  |
|  | If I get vaccinated, I protect myself from suffering | | *1,166* |  | *1,166* |  | *1,166* |  |
|  |  | Do not agree | 80 | 6.9 | 58 | 5.0 | 90 | 7.7 |
|  |  | Agree | 915 | 78.5 | 919 | 78.8 | 1,050 | 90.1 |
|  |  | Don’t know | 171 | 14.7 | 189 | 16.2 | 26 | 2.2 |
|  | If I get vaccinated, I protect my family members | | *1,165* |  | *1,166* |  | *1,165* |  |
|  |  | Do not agree | 261 | 22.4 | 213 | 18.3 | 220 | 18.9 |
|  |  | Agree | 753 | 64.6 | 754 | 64.7 | 915 | 78.5 |
|  |  | Don’t know | 151 | 13.0 | 199 | 17.1 | 30 | 2.6 |
| **Vaccine barriers (HZ, RSV, I)** | | |  |  |  |  |  |  |
|  | If I get vaccinated, it could hurt me | | *1,166* |  | *1,166* |  | *1,165* |  |
|  |  | Do not agree | 545 | 46.7 | 511 | 43.8 | 817 | 70.1 |
|  |  | Agree | 192 | 16.5 | 200 | 17.2 | 236 | 20.3 |
|  |  | Don’t know | 429 | 36.8 | 455 | 39.0 | 112 | 9.6 |
|  | It's too complicated to get the vaccine | | *1,166* |  | *ND* |  | *1,166* |  |
|  |  | Do not agree | 212 | 18.2 |  |  | 900 | 77.2 |
|  |  | Agree | 570 | 48.9 |  |  | 229 | 19.6 |
|  |  | Don’t know | 384 | 32.9 |  |  | 37 | 3.2 |
| **Action on the vaccine (****HZ, RSV, I)** | | |  |  |  |  |  |  |
|  | I would get vaccinated if… | |  |  |  |  |  |  |
|  | A family member/partner recommends it | | *1,166* |  | *1,166* |  | *1,166* |  |
|  |  | Do not agree | 254 | 21.8 | 304 | 26.1 | 246 | 21.1 |
|  |  | Agree | 790 | 67.8 | 706 | 60.5 | 900 | 77.2 |
|  |  | Don’t know | 122 | 10.5 | 156 | 13.4 | 20 | 1.7 |
|  | An acquaintance recommends it | | *1,166* |  | *1,166* |  | *1,166* |  |
|  |  | Do not agree | 592 | 50.8 | 627 | 53.8 | 573 | 49.1 |
|  |  | Agree | 429 | 36.8 | 374 | 32.1 | 543 | 46.6 |
|  |  | Don’t know | 145 | 12.4 | 165 | 14.2 | 50 | 4.3 |
|  | A health professional recommends it | | *1,166* |  | *1,166* |  | *1,166* |  |
|  |  | Do not agree | 39 | 3.3 | 32 | 2.7 | 38 | 3.3 |
|  |  | Agree | 1,069 | 91.7 | 1,053 | 90.3 | 1,115 | 95.6 |
|  |  | Don’t know | 58 | 5.0 | 81 | 6.9 | 13 | 1.1 |
|  | The government recommends it | | *1,166* |  | *1,166* |  | *1,166* |  |
|  |  | Do not agree | 216 | 18.5 | 217 | 18.6 | 192 | 16.5 |
|  |  | Agree | 796 | 68.3 | 778 | 66.7 | 919 | 78.8 |
|  |  | Don’t know | 154 | 13.2 | 171 | 14.7 | 55 | 4.7 |
| **Safety/effectiveness of the vaccine** | | |  |  |  |  |  |  |
|  | How do you consider the vaccine (HZ, RSV, I) | |  |  |  |  |  |  |
|  | Effective (it works) | | *1,166* |  | *1,166* |  | *1,166* |  |
|  |  | A lot | 185 | 15.9 | 188 | 16.1 | 817 | 70.1 |
|  |  | A little | 119 | 10.2 | 113 | 9.7 | 238 | 20.4 |
|  |  | Not | 19 | 1.6 | 18 | 1.5 | 9 | 0.8 |
|  |  | Don’t know | 843 | 72.3 | 847 | 72.6 | 102 | 8.7 |
|  | Safe (reliable) | | *1,166* |  | *1,166* |  | *1,166* |  |
|  |  | A lot | 203 | 17.4 | 194 | 16.6 | 822 | 70.5 |
|  |  | A little | 126 | 10.8 | 130 | 11.1 | 238 | 20.4 |
|  |  | Not | 28 | 2.4 | 23 | 2.0 | 11 | 0.9 |
|  |  | Don’t know | 809 | 69.4 | 819 | 70.2 | 95 | 8.1 |
| **Social norm, vaccination (HZ, RSV, I)** | | |  |  |  |  |  |  |
|  | If others get vaccinated, I would get vaccinated | | *1,166* |  | *1,166* |  | *1,166* |  |
|  |  | Do not agree | 290 | 24.9 | 298 | 25.6 | 273 | 23.4 |
|  |  | Agree | 650 | 55.7 | 620 | 53.2 | 834 | 71.5 |
|  |  | Don’t know | 226 | 19.4 | 248 | 21.3 | 59 | 5.1 |
| **Vaccination intention (HZ, RSV, I)** | | |  |  |  |  |  |  |
|  | If I am offered to get vaccinated, I would do it | | *1,166* |  | *1,166* |  | *1,166* |  |
|  |  | Do not agree | 211 | 18.1 | 184 | 15.8 | 148 | 12.7 |
|  |  | Agree | 701 | 60.1 | 705 | 60.5 | 965 | 82.8 |
|  |  | Don’t know | 254 | 21.8 | 277 | 23.8 | 53 | 4.5 |
|  | I would recommend the vaccine | | *ND* |  | *ND* |  | *1,165* |  |
|  |  | Do not agree |  |  |  |  | 154 | 13.2 |
|  |  | Agree |  |  |  |  | 959 | 82.3 |
|  |  | Don’t know |  |  |  |  | 52 | 4.5 |

In italics, the number of participants who were asked the question.

* Not all variables sum to 100% due to missing data.

n, number; ND, not determined (not asked); RSV, respiratory syncytial virus.
