# Supplementary material for: Knowledge, attitudes, and practices related to adult vaccination among adults and healthcare professionals across Mexico
Source: PLoS One. 2026 May 21;21(5):e0348625. doi: 10.1371/journal.pone.0348625 (PMC13193386; doi:10.1371/journal.pone.0348625)
Supplement: S3 Table — (DOCX) [file pone.0348625.s004.docx]

**S3 Table. Practices regarding new vaccines among the general population**

|  |  | **New vaccinations/national health card** | **n** | **%*** |
| --- | --- | --- | --- | --- |
|  |  | Total number | ***1,169*** |  |
| **Practices regarding new vaccines** | | |  |  |
|  | Would take a new vaccine | | *1,169* |  |
|  |  | Very likely | 337 | 28.8 |
|  |  | Not very likely | 527 | 45.1 |
|  |  | Not likely | 132 | 11.3 |
|  |  | Don’t know | 173 | 14.8 |
|  | What information do you need to accept it? | | *1,168* |  |
|  |  | About the disease it prevents | 507 | 43.4 |
|  |  | About the complications it prevents | 149 | 12.8 |
|  |  | About safety/adverse effects | 167 | 14.3 |
|  |  | About whether the vaccine works | 299 | 25.6 |
|  |  | Other | 46 | 3.9 |
|  | If you are offered the RSV vaccine, would you accept it? | | *1,168* |  |
|  |  | Very likely | 374 | 32.0 |
|  |  | Not very likely | 508 | 43.5 |
|  |  | Not likely | 142 | 12.2 |
|  |  | Don’t know | 144 | 12.3 |
|  | If you are offered the herpes zoster vaccine, would you accept it? | | *1,168* |  |
|  |  | Very likely | 377 | 32.3 |
|  |  | Not very likely | 497 | 42.6 |
|  |  | Not likely | 146 | 12.5 |
|  |  | Don’t know | 148 | 12.7 |
| **Information on the national health card** | | |  |  |
|  | Has a card | | *1,167* |  |
|  |  | You have it and have information | 50 | 4.3 |
|  |  | You have it, but didn't show it | 609 | 52.2 |
|  |  | You don’t have it | 494 | 42.3 |
|  |  | You showed it but don’t have information | 14 | 1.2 |
|  | Why don't you have a card? | | *494* |  |
|  |  | Never had it | 122 | 24.7 |
|  |  | Lost it | 54 | 10.9 |
|  |  | Didn’t bring it | 290 | 58.7 |
|  |  | It deteriorated | 19 | 3.8 |
|  |  | Decided not to show it | 9 | 1.8 |

*The percentages do not all add up to 100% due to missing data. In italics, the number of participants who were asked the question.

n, number; RSV, respiratory syncytial virus.
