# Supplementary material for: Knowledge, attitudes, and practices related to adult vaccination among adults and healthcare professionals across Mexico
Source: PLoS One. 2026 May 21;21(5):e0348625. doi: 10.1371/journal.pone.0348625 (PMC13193386; doi:10.1371/journal.pone.0348625)
Supplement: S4 Table — (DOCX) [file pone.0348625.s005.docx]

**S4 Table. Knowledge and perceptions among HCPs (N = 228)**

|  |  |  | **Whooping cough (W)** | | **Influenza (I)** | | **Herpes zoster (HZ)** | | **RSV** | |
| --- | --- | --- | --- | --- | --- | --- | --- | --- | --- | --- |
|  |  |  | **n** | **%*** | **n** | **%*** | **n** | **%*** | **n** | **%*** |
| **Cases treated/vaccinated among adults** | | | |  |  |  |  |  |  |  |
|  | Treated cases: | | *226* |  | *226* |  | *226* |  | *225* |  |
|  |  | No | 206 | 91.2 | 97 | 42.9 | 144 | 63.7 | 171 | 76 |
|  |  | Yes | 20 | 8.8 | 126 | 55.8 | 82 | 36.3 | 50 | 22.2 |
|  |  | Don’t know | 0 | 0.0 | 3 | 1.3 | 0 | 0.0 | 4 | 1.8 |
|  | Got vaccinated against the disease. last 5 years: | | *226* |  | *226* |  | *226* |  | *ND* |  |
|  |  | No | 124 | 54.9 | 3 | 1.3 | 204 | 90.3 |  |  |
|  |  | Yes | 101 | 44.7 | 223 | 98.7 | 17 | 7.5 |  |  |
|  |  | Don’t know | 1 | 0.4 | 0 | 0.0 | 5 | 2.2 |  |  |
|  | Vaccination services at your workplace: | | *226* |  | *226* |  | *226* |  | *ND* |  |
|  |  | No | 32 | 14.2 | 14 | 6.2 | 185 | 81.9 |  |  |
|  |  | Yes | 184 | 81.4 | 212 | 93.8 | 20 | 8.8 |  |  |
|  |  | Don’t know | 10 | 4.4 | 0 | 0.0 | 21 | 9.3 |  |  |
|  | On occasion recommended vaccinating older adults: | | *226* |  | *226* |  | *226* |  | *ND* |  |
|  |  | No | 108 | 47.8 | 9 | 4.0 | 170 | 75.2 |  |  |
|  |  | Yes | 118 | 52.2 | 217 | 96.0 | 53 | 23.5 |  |  |
|  |  | Don’t know | 0 | 0.0 | 0 | 0.0 | 3 | 1.3 |  |  |
|  | Usually recommends the vaccine: | | *226* |  | *226* |  | *226* |  | *ND* |  |
|  |  | No | 54 | 23.9 | 5 | 2.2 | 142 | 62.8 |  |  |
|  |  | Yes | 170 | 75.2 | 221 | 97.8 | 78 | 34.5 |  |  |
|  |  | Don’t know | 2 | 0.9 | 0 | 0.0 | 6 | 2.7 |  |  |
|  | Recommended vaccination schedule (W) | | *226* |  |  |  |  |  |  |  |
|  |  | Pregnant women. 1 dose per pregnancy | 57 | 25.2 |  |  |  |  |  |  |
|  |  | Adult women and men. 1 dose every 10 years | 49 | 21.7 |  |  |  |  |  |  |
|  |  | Pregnant women/Adult women and men | 103 | 45.6 |  |  |  |  |  |  |
|  |  | None | 17 | 7.5 |  |  |  |  |  |  |
|  | Recommended vaccination schedule (I) | |  |  | *226* |  |  |  |  |  |
|  |  | 1 annual dose in winter for at-risk groups | | | 207 | 91.6 |  |  |  |  |
|  |  | < 5 years |  |  | 6 | 2.7 |  |  |  |  |
|  |  | Pregnancy |  |  | 1 | 0.4 |  |  |  |  |
|  |  | Chronically ill |  |  | 1 | 0.4 |  |  |  |  |
|  |  | Healthcare personnel |  |  | 5 | 2.2 |  |  |  |  |
|  |  | Older adults |  |  | 6 | 2.7 |  |  |  |  |
|  | Recommended vaccination schedule (HZ) | |  |  |  |  | *226* |  |  |  |
|  |  | 1 dose |  |  |  |  | 50 | 22.1 |  |  |
|  |  | 2 doses |  |  |  |  | 55 | 24.3 |  |  |
|  |  | Don’t remember |  |  |  |  | 121 | 53.5 |  |  |
| **Perception of disease priority (W, I, HZ, RSV)** | | | |  |  |  |  |  |  |  |
|  | It is a substantial disease burden | | *226* |  | *226* |  | *226* |  | *226* |  |
|  |  | Do not agree | 71 | 31.4 | 46 | 20.4 | 103 | 45.6 | 48 | 21.2 |
|  |  | Agree | 143 | 63.3 | 178 | 78.8 | 100 | 44.2 | 148 | 65.5 |
|  |  | Don’t know | 12 | 5.3 | 2 | 0.9 | 23 | 10.2 | 30 | 13.3 |
|  | It has a significant impact on the general population | | *226* |  | *226* |  | *226* |  | *ND* |  |
|  |  | Do not agree | 36 | 15.9 | 17 | 7.5 | 60 | 26.5 |  |  |
|  |  | Agree | 185 | 81.9 | 209 | 92.5 | 152 | 67.3 |  |  |
|  |  | Don’t know | 5 | 2.2 | 0 | 0.0 | 14 | 6.2 |  |  |
|  | It has a significant impact on children | | *ND* |  | *ND* |  | *ND* |  | *226* |  |
|  |  | Do not agree |  |  |  |  |  |  | 5 | 2.2 |
|  |  | Agree |  |  |  |  |  |  | 200 | 88.5 |
|  |  | Don’t know |  |  |  |  |  |  | 21 | 9.3 |
|  | It is a high priority | | *226* |  | *226* |  | *226* |  | *ND* |  |
|  |  | Do not agree | 52 | 23.0 | 24 | 10.6 | 105 | 46.5 |  |  |
|  |  | Agree | 168 | 74.3 | 201 | 88.9 | 113 | 50.0 |  |  |
|  |  | Don’t know | 6 | 2.7 | 1 | 0.4 | 8 | 3.5 |  |  |
|  | It has a significant impact on adults | | *226* |  | *226* |  | *226* |  | *226* |  |
|  |  | Do not agree | 27 | 11.9 | 3 | 1.3 | 40 | 17.7 | 24 | 10.6 |
|  |  | Agree | 198 | 87.6 | 223 | 98.7 | 176 | 77.9 | 179 | 79.2 |
|  |  | Don’t know | 1 | 0.4 | 0 | 0.0 | 10 | 4.4 | 23 | 10.2 |
|  | Greater risk of severity in older adults | | *226* |  | *226* |  | *226* |  | *226* |  |
|  |  | Do not agree | 24 | 10.6 | 8 | 3.5 | 54 | 23.9 | 29 | 12.8 |
|  |  | Agree | 195 | 86.3 | 217 | 96.0 | 159 | 70.4 | 180 | 79.6 |
|  |  | Don’t know | 7 | 3.1 | 1 | 0.4 | 13 | 5.8 | 17 | 7.5 |
| **Perception of vaccine safety (W, I, HZ)** | | |  |  |  |  |  |  |  |  |
|  | It is considered safe for older adults | | *226* |  | *226* |  | *226* |  | *ND* |  |
|  |  | Do not agree | 22 | 9.7 | 3 | 1.3 | 10 | 4.4 |  |  |
|  |  | Agree | 190 | 84.1 | 222 | 98.2 | 155 | 68.6 |  |  |
|  |  | Don’t know | 14 | 6.2 | 1 | 0.4 | 61 | 27.0 |  |  |
| **Perception of vaccine efficacy (W, I, HZ)** | | |  |  |  |  |  |  |  |  |
|  | It is considered effective for adults | | *226* |  | *226* |  | *226* |  | *ND* |  |
|  |  | Do not agree | 15 | 6.6 | 2 | 0.9 | 12 | 5.3 |  |  |
|  |  | Agree | 205 | 90.7 | 224 | 99.1 | 190 | 84.1 |  |  |
|  |  | Don’t know | 6 | 2.7 | 0 | 0.0 | 24 | 10.6 |  |  |
| **Awareness. Recommendations of the SS** | | |  |  |  |  |  |  |  |  |
|  | Your institute recommends vaccinating older adults | | *226* |  | *226* |  | *226* |  | *ND* |  |
|  |  | Do not agree | 51 | 22.6 | 3 | 1.3 | 101 | 44.7 |  |  |
|  |  | Agree | 159 | 70.4 | 223 | 98.7 | 84 | 37.2 |  |  |
|  |  | Don’t know | 16 | 7.1 | 0 | 0.0 | 41 | 18.1 |  |  |
|  | Clear recommendations: Vaccinate older adults | | *ND* |  | *226* |  | *226* |  | *ND* |  |
|  |  | Do not agree |  | - | 3 | 1.3 | 51 | 22.6 |  |  |
|  |  | Agree |  | - | 222 | 98.2 | 118 | 52.2 |  |  |
|  |  | Don’t know |  | - | 1 | 0.4 | 57 | 25.2 |  |  |

In italics, the number of participants who were asked the question.

*The percentages do not add up to 100% due to missing data.

HCP, healthcare professional; N, number; n, number; ND, not determined (not asked); RSV, respiratory syncytial virus; SS, Ministry of Health (Secretaría de Salud).
